# STAT3 mutations are frequent in T-cell large granular lymphocytic leukemia with pure red cell aplasia

**DOI:** 10.1186/1756-8722-6-82

**Published:** 2013-10-31

**Authors:** Zhi-Yuan Qiu, Lei Fan, Li Wang, Chun Qiao, Yu-Jie Wu, Jian-Feng Zhou, Wei Xu, Jian-Yong Li

**Affiliations:** 1 Department of Hematology, the First Affiliated Hospital of Nanjing Medical University, Jiangsu Province Hospital, Nanjing 210029, China; 2 Department of Hematology, Tongji Medical College, Huazhong University of Science and Technology, Tongji Hospital, Wuhan, China

**Keywords:** STAT3, T-LGLL, PRCA, β2-MG

## Abstract

T-cell large granular lymphocytic leukemia (T-LGLL) is a rare lymphoproliferative disorder and can cooccur in the context of pure red cell aplasia (PRCA). The aim of the current study was to analyze the signal transducer and activator of transcription 3 (STAT3) mutation status and its clinical significance in T-LGLL. We found STAT3 mutations in 21.4% of patients with T-LGLL. High β2-MG (β2-microglobulin) levels (*P* = 0.005), neutropenia (*P* = 0.018) and PRCA (*P* = 0.001) all displayed a significant association with STAT3 mutations. In univariate analysis, treatment-free survival (TFS) was affected by STAT3 mutation status (*P* = 0.008) and β2-MG (*P* = 0.006). Our results demonstrate the remarkable correlation of STAT3 mutation with PRCA, neutropenia and β2-MG.

## To the editor

T-cell large granular lymphocytic leukemia (T-LGLL) is a rare lymphoproliferative disorder and can cooccur in the context of pure red cell aplasia (PRCA) [1-3]. Recently, recurrent somatic mutations in the Src homology domain of the signal transducer and activator of transcription 3 (STAT3) gene have been identified to have a high frequency of 40% [4] and 33% [5] in T-LGLL. To analyze the STAT3 mutation status and its clinical significance, we investigated STAT3 mutations in 28 consecutive patients with newly diagnosed T-LGLL who were recruited between January 2007 and January 2013. The diagnosis of T-LGLL was based on the WHO criteria [1]. The diagnosis of PRCA was defined according to the previous report [6].

## Findings

For STAT3 mutation screening, genes of exons 20 and 21 of STAT3 were amplified by PCR and sequenced. Five different mutations (Y640F, D661Y, E616V, V671F, S614R) were observed, and two mutations, E616V and V671F, had not been previously reported. STAT3 is an oncogene, and its activation plays a key role in cell signaling in many types of cancer [7]. In our study, all mutations were heterozygous and the mutational hot spot were located close to the transcriptional activation domain.

Seven patients (25%) were found to have both T-LGLL and PRCA. STAT3 mutation was more common among patients with PRCA than those without PRCA (71.4% vs.4.8%, *P* = 0.001). Six of 7 (85.7%) patients with PRCA were found to have elevated β2-MG (β2-microglobulin), which was significantly higher than was found in 6 of 18 (33.3%) patients without PRCA (*P* = 0.030, Table 1). On the other hand, patients with STAT3 mutations had presented with neutropenia more often than those without STAT3 mutations (100% vs. 40.9%, *P* = 0.018), and this is similar to previous studies [4].

**Table 1:** Comparison of clinical characteristics between T-LGLL patients with or without PRCA

	**Patients with PRCA (7)**	**Patients without PRCA (21)**	***P***
Gender			1.000
Male	3 (42.9)	8 (38.1)	
Female	4 (57.1)	13 (61.9)	
Age			0.668
Mean ± SD	57.3 ± 10.2	55.4 ± 9.0	
LDH			0.165
>250 U/L	4 (57.1)	5 (23.8)	
<250 U/L	3 (42.9)	16 (76.2)	
β2-MG (n = 25)			0.030
>3.0 mg/L	6 (85.7)	6 (33.3)	
<3.0 mg/L	1 (14.3)	12 (66.7)	
Neutropenia, no.%	6 (85.7)	9 (42.9)	0.084
Lymphocytosis, no.%	4 (57.1)	11 (52.4)	1.000
LGL count in PB,×10^9^/L			0.295
Mean ± SD	3.2 ± 2.1	2.9 ± 1.7	
Splenomegaly, no.%	5 (71.4)	5 (23.8)	0.063
STAT3 mutation	5 (71.4)	1 (4.8)	0.001

PB: peripheral blood; STAT3: signal transducer and activator of transcription 3; Anemia:hemoglobin (Hb) <100 g/L; Neutropenia: absolute neutrophil count (ANC) <1.5 × 10^9^/L.

LDH: lactic dehydrogenase.

Anemia, neutropenia and rheumatoid arthritis (RA) are common complications, and anemia is more common in Asian countries [8,9]; neutropenia and RA is more common in Western countries [10,11], but there was no patients with RA in our study. We show here that the coexistence of PRCA or neutropenia is more frequent in patients with STAT3 mutation. This observation varies from from the study of Jerez et al. [5] and Koskela et al. [4], but is consistent with the study from Japan [12].

**Figure 1:**
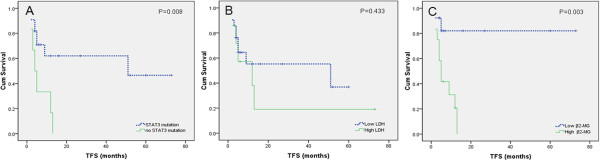
**TFS according to the STAT3 mutation status, serum LDH levels and serum β2-MG levels determined at diagnosis.** Low LDH group: <250 U/L, and high LDH group: >250 U/L. Low β2-MG group: <3.0 mg/L, and high β2-MG group: >3.0 mg/L. Analysis identified that the shorter TFS in the STAT3 mutation group **(A)** and high β2-MG group **(C)**, but TFS was not related to LDH levels **(B)**.

TFS was defined as the period from the diagnosis date to the time of the first treatment. In our study, we observed a significant difference between patients with or without STAT3 mutations in TFS (median 6.5 months vs. 16.6 months, *P* = 0.008, Figure 1A), and we observed a significant difference between the high β2-MG group and the low β2-MG group in TFS (P = 0.003 Figure 1C). TFS was not related to LDH levels (Figure 1B).

To our knowledge, our study is the first report on STAT3 mutation status in patients with T-LGLL in China. Although the STAT3 mutation thus likely contributes to the pathogenesis of T-LGLL, patients without STAT3 mutations are characterized by significant heterogeneity, indicating that other mechanisms of STAT3 activation can be operative in this disease. Further studies are therefore necessary to determine other reasons to lead to the pathogenesis of T-LGLL.

## Abbreviations

T-LGLL: T-cell large granular lymphocytic leukemia; STAT3: Signal transducer and activator of transcription 3; PRCA: Pure red blood cell aplasia; LDH: Lactic dehydrogenase; β2-MG: β2-microglobulin; TFS: Treatment-free survival; PB: Peripheral blood; ANC: Absolute neutrophil count; RA: Rheumatoid arthritis.

## Competing interests

The authors declare that they have no competing interests.

## Authors’ contributions

ZYQ performed the laboratory work for this study and wrote the manuscript; LF and LW provided material and clinical information; CQ and YJW designed the experiments; JFZ analyzed data; WX and JYL performed statistical analysis and wrote the manuscript. All authors have approved the final version of the manuscript.
